# Metformin Represses Self-Renewal of the Human Breast Carcinoma Stem Cells via Inhibition of Estrogen Receptor-Mediated OCT4 Expression

**DOI:** 10.1371/journal.pone.0028068

**Published:** 2011-11-23

**Authors:** Ji-Won Jung, Sang-Bum Park, Soo-Jin Lee, Min-Soo Seo, James E. Trosko, Kyung-Sun Kang

**Affiliations:** 1 Department of Veterinary Public Health, College of Veterinary Medicine, Adult Stem Cell Research Center, Seoul National University, Seoul, Republic of Korea; 2 Department of Pediatrics and Human Development, College of Human Medicine, Center for Integrative Toxicology, Michigan State University, East Lansing, Michigan, United States of America; Health Canada, Canada

## Abstract

Metformin, a Type II diabetic treatment drug, which inhibits transcription of gluconeogenesis genes, has recently been shown to lower the risk of some diabetes-related tumors, including breast cancer. Recently, “cancer stem cells” have been demonstrated to sustain the growth of tumors and are resistant to therapy. To test the hypothesis that metformin might be reducing the risk to breast cancers, the human breast carcinoma cell line, MCF-7, grown in 3-dimensional mammospheres which represent human breast cancer stem cell population, were treated with various known and suspected breast cancer chemicals with and without non-cytotoxic concentrations of metformin. Using OCT4 expression as a marker for the cancer stem cells, the number and size were measured in these cells. Results demonstrated that TCDD (100 nM) and bisphenol A (10 µM) increased the number and size of the mammospheres, as did estrogen (10 nM E2). By monitoring a cancer stem cell marker, OCT4, the stimulation by these chemicals was correlated with the increased expression of OCT4. On the other hand, metformin at 1 and 10 mM concentration dramatically reduced the size and number of mammospheres. Results also demonstrated the metformin reduced the expression of OCT4 in E2 & TCDD mammospheres but not in the bisphenol A mammospheres, suggesting different mechanisms of action of the bisphenol A on human breast carcinoma cells. In addition, these results support the use of 3-dimensional human breast cancer stem cells as a means to screen for potential human breast tumor promoters and breast chemopreventive and chemotherapeutic agents.

## Introduction

Metformin, a Type 2 diabetic treatment drug, which inhibits transcription of gluconeogenesis genes [1], has recently been shown to lower the risk of some diabetes-related tumors, including breast cancer [2]–[15]. However, not all studies demonstrate this response [2] possibly due to confounding factors. Although patients with diabetes are at high risk for cancers of the liver, pancreas, endometrium, breast, colon, and bladder, it is not clear as to whether the positive effects of metformin against certain cancers affects the cancer, directly or indirectly, by inhibiting the diabetic state. In addition, it is not clear whether metformin might affect other cancers in non-diabetic individuals. Moreover, metformin inhibited the growth of breast cancer cell lines in vitro. However, in some cases, it inhibited non-transformed cells at similar concentrations [16]–[18].

Recently, it has been demonstrated that “cancer stem cells” sustain the growth of tumors and are resistant to therapy. MCF-7 mammospheres have been shown to enrich breast cancer stem cells expressing CD44^+^CD24^−/low^ [19], [20]. Assuming the concept of “cancer stem cells” as the “tumor-initiating” or “tumor-sustaining” cells of any tumor or permanent cell line [21]–[23], the objective of this study was to determine the effects of several known epigenetic-acting chemicals, such as endocrine disrupting- or tumor promoting chemicals (phenol red [24], TCDD [25], [26] and bisphenol A [27]), compared to estrogen's effect on the growth of MCF-7 mammospheres. These chemical –treated mammospheres were exposed to metformin at various non-cytotoxic concentrations. In effect, this series of experiments was designed to test the hypothesis that metformin might be reducing the risk to certain cancers by affecting the breast cancer stem cells in these mammospheres.

The results, in general, demonstrated that metformin reduced the expression of Oct4 in E2- and TCDD- treated human breast cancer stem cells in MCF-7 mammospheres, but not in the bisphenol A-treated mammospheres, suggesting a different mechanism of action of the bisphenol A on the breast cancer stem cells self-renewal ability. In addition, the study supports the use of 3-dimensional mammospheres to screen for potential human breast tumor promoters or cancer chemopreventive or chemotherapeutic agents.

## Results

### The mammosphere formations of human breast cell lines

**Figure 1:**
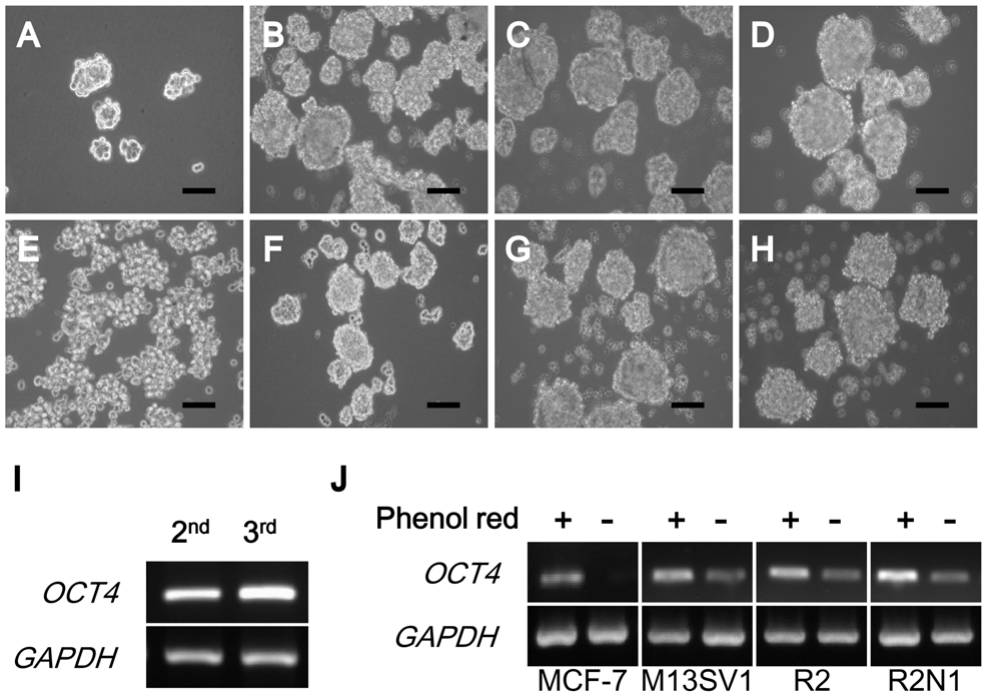
ER positive (A–D and F–H) and negative (E) human breast cells in phenol red-contained (A–E) or phenol red-free MEBM (F–H), expression level of *OCT4* mRNA in passaged MCF-7 mammospheres (I), and several ER+ breast cancer mammospheres cultured in MEBM with or without phenol red (J). ; (A) MCF-7; (B), (F) M13SV1; (C), (G) M13SV1 R2; (D), (H) M13SV1 R2N1; (E) MDA-MB-231. The magnification was X 200. Scale bar represents 50 µm in length.

The mammospheres were generated from the ERα positive human breast cancer cell line, MCF-7, M13SV1, M13SV1 R2 and M13SV1 R2N1, in phenol red-containing MEBM and phenol red-free MEBM. In both media, the cells efficiently formed compact mammospheres (Figure 1). MCF-7 cells were continuously capable of forming mammospheres through repeated subcultures in medium with phenol red (data not shown). ER- negative human breast cancer cell lines, MDA-MB-231 cells (Figure 1E) and SK-BR-3 cells (data not shown), failed to form mammospheres in both phenol red-contained MEBM and phenol red-free MEBM. Rather, they formed aggregated clusters of cells. It suggests that the estrogen receptor status of breast cells affected the formation and maintenance of mammospheres.

### Flow cytometric analysis of MCF-7 mammospheres

As stated above, MCF-7 cells efficiently formed mammospheres and this ability was maintained through repeated subcultures in phenol red-contained media. To identify the relationship of mammosphere formation and cancer stem cell population, we carried out flow cytometry using the cancer stem cell markers (CD44^+^/ CD24^−/low^) [28]. The results indicated that secondary mammospheres consisted of 0.1% (through side scatter; P1) and 2.7% (through forward scatter; P2) mammary stem cell population, while tertiary mammospheres had 1.1% (P1) and 15.9% (P2). Indeed, as mammospheres were passaged, cancer stem cell populations were increased. The mRNA expression of *OCT4* gene was up-regulated in tertiary mammospheres compared to secondary mammospheres (Figure 1I).

### OCT4 expression induced by phenol red in mammospheres

Phenol red has been shown to act as a weak estrogens in ER- positive MCF-7 cell line [24]. In order to examine the effects of phenol red on the stemness of ER-positive human mammospheres (MCF-7, M13SV1, M13SV1 R2, M13SV1 R2N1), we measured the cancer stem cell marker, *OCT4* gene expression, in mammospheres cultured in phenol red-free or phenol red-containing MEBM. In most cases, where the mammospheres were cultured in phenol red-free MEBM, *OCT4* gene expression was significantly decreased compared to phenol red-containing medium (Figure 1J). Therefore, it was suggested that estrogenicity does have a role in *OCT4* expression in ER-responsive human breast cells.

### 17-beta-estradiol induced OCT4 expression in MCF-7 mammospheres

**Figure 2:**
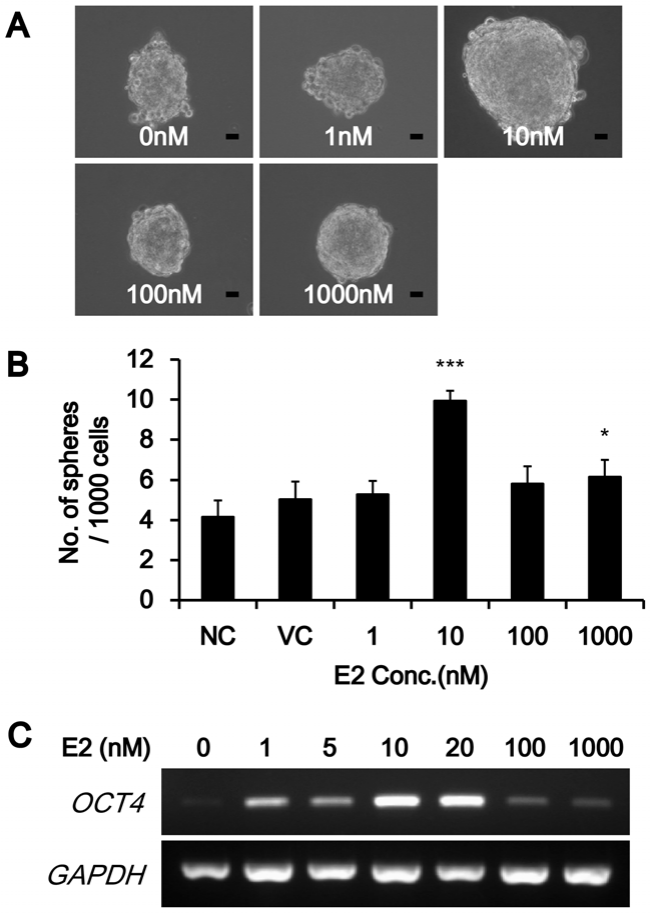
Effect of E2 on MCF-7 mammospheres. (A), (B) Mammosphere formation was increased by 10 nM E2 treatment. Data were presented as the number of mammospheres per 1,000 seeded cells at 5d (mean ± SD., n = 3). The magnification was X 200. Scale bar represents 10 µm in length. *, *P*<0.05; ***, *P*<0.001. (C) 10 nM and 20 nM E2 induced *OCT4* expression dramatically in RT-PCR.

To identify the direct relationship between mammosphere formation and estrogen, we treated of 17-beta-estradiol (E2) in MCF-7 mammospheres (1 nM to 1000 nM). Mammospheres of the biggest size and of the largest in number were observed at 10 nM concentration of E2 (Figures 2A, B). Interestingly, the highest level of OCT4 expression was observed at 10 nM concentration of E2 (Figure 2C) as well. Therefore, 10 to 20 nM concentration of E2 could induce dramatic increase of *OCT4* expression and proliferation of mammospheres, as well as the breast cancer stem cell population in MCF-7 mammospheres.

### ER antagonist inhibits estrogen-induced mammosphere formation and OCT4 expression

**Figure 3:**
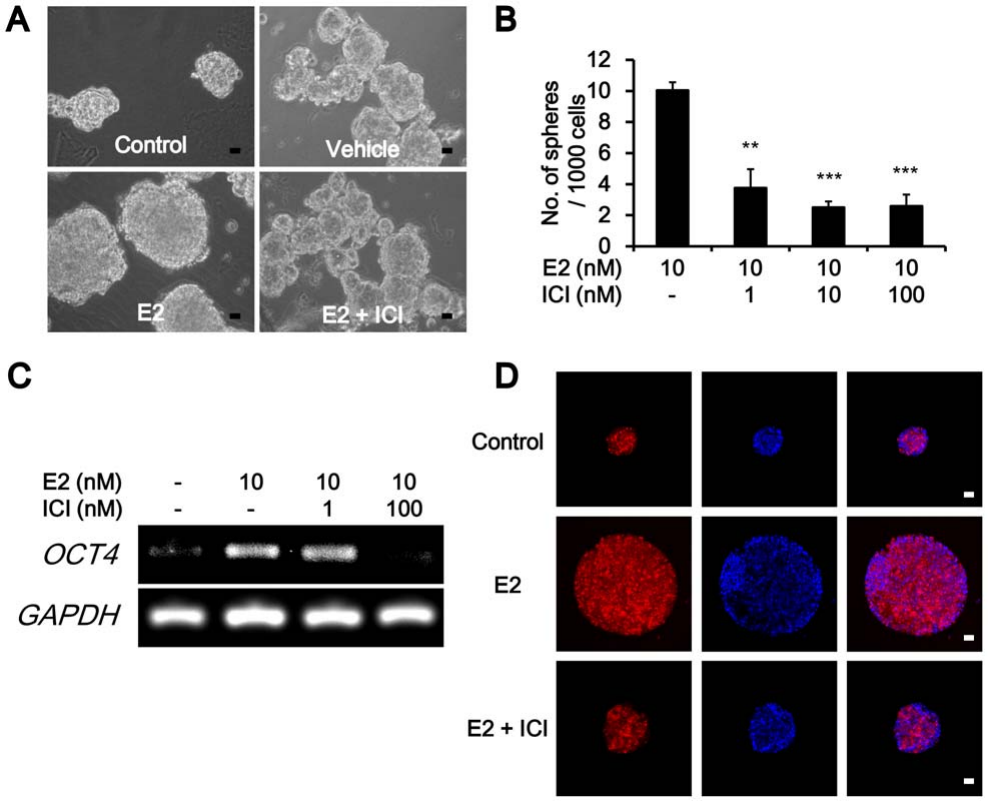
Direct pathways of E2 on MCF-7 mammosphere proliferation. (A) 100 nM ICI 182,780 suppressed the effect of E2 on increment of the size and number of mammospheres. The magnification was X 200. Scale bar represents 10 µm in length. (B) Number of mammospheres was decreased by co-treatment with ICI 182,780. Data were presented as the number of mammospheres per 1,000 seeded cells at 5d (mean ± S.D., n = 3). **, *P*<0.01; ***, *P*<0.001. (C) 100 nM ICI 182,780 could repress the *OCT4* induction by 10 nM E2. (D) Immunocytochemical detection of OCT4 in MCF-7 mammospheres, compared to the non-treated control group (Control), 10 nM E2-treated (E2), and 100 nM ICI 182,780 with 10 nM E2 (E2 + ICI). The magnification was X 200. Scale bar represents 10 µm in length.

To confirm whether the above-mentioned effect of estrogen was ER dependent, we treated the MCF-7 cells with the ER alpha antagonist, ICI 182,780, along with 17-beta-estradiol. The results showed that the size and number of mammospheres were decreased when they were co-treated with 10 nM E2 and 100 nM ICI 182,780 (Figures 3A, B). Interestingly, these inhibitory effects of ER alpha antagonist occurred, not only on mammosphere formation, but also on *OCT4* expression. ICI 182,780 repressed *OCT4* induction by estrogen (Figures 3C, D). These results suggest that low concentration E2 (1–20 nM) might up-regulate *OCT4* expression through an ER-dependent pathway.

### Effect of the antioxidant in high concentration of E2

**Figure 4:**
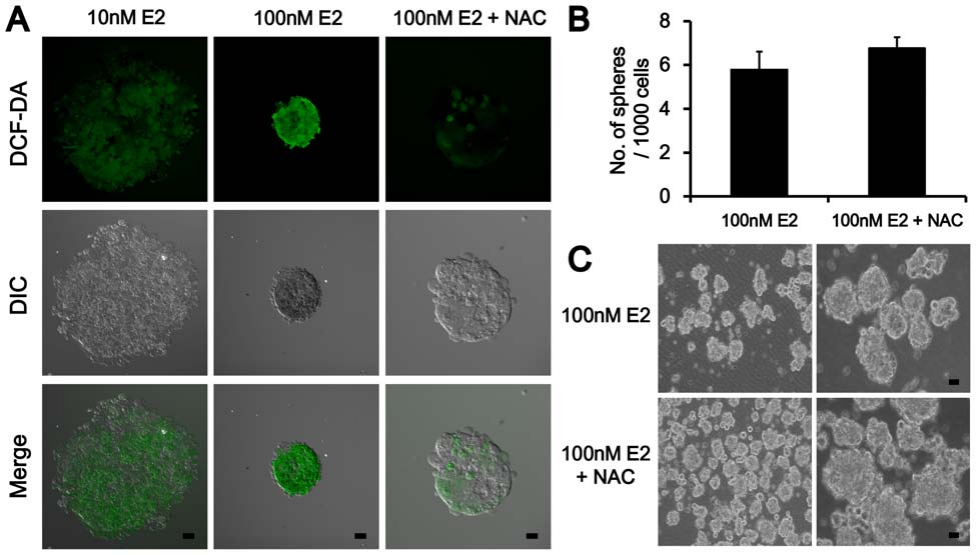
Indirect pathways of E2 on MCF-7 mammosphere proliferation. (A) ROS detection with DCF-DA. MCF-7 mammospheres treated with 10 nM E2 (left), 100 nM E2 (center), or in concert with 10 µM NAC for 7d (right). The magnification was X 200. Scale bar represents 10 µm in length. (B) The change of mammosphere number by co-treatment with 10 µM NAC. Results were expressed as the number of mammospheres per 1,000 seeded cells at 5d (mean ± SD, n = 3). (C) Secondary MCF-7 mammospheres treated with 100 nM E2 (upper panels) in concert with 10 µM NAC (lower panels). The original magnifications were X 200 except left panel of (C) (X 100). Scale bar represents 10 µm in length.

Estrogen was reported to induce reactive oxygen species (ROS) production in high concentration [29], [30]. MCF-7 mammospheres were treated with N-acetyl-L-cysteine (NAC), 10 µM with high concentration E2 (100 nM). Interestingly, NAC increased the size, number of primary mammospheres and reduced the ROS production levels (Figures 4A, B). Moreover, the number of secondary mammospheres was increased (Figure 4C). These results might suggest 10 µM NAC blocks E2-induced oxidative stress and subsequent cell damage to increase proliferation and symmetrical cell division of the OCT4 positive cancer stem cells in MCF-7 mammospheres.

### Regulation of mammosphere formation by breast cancer promoters and metformin

**Figure 5:**
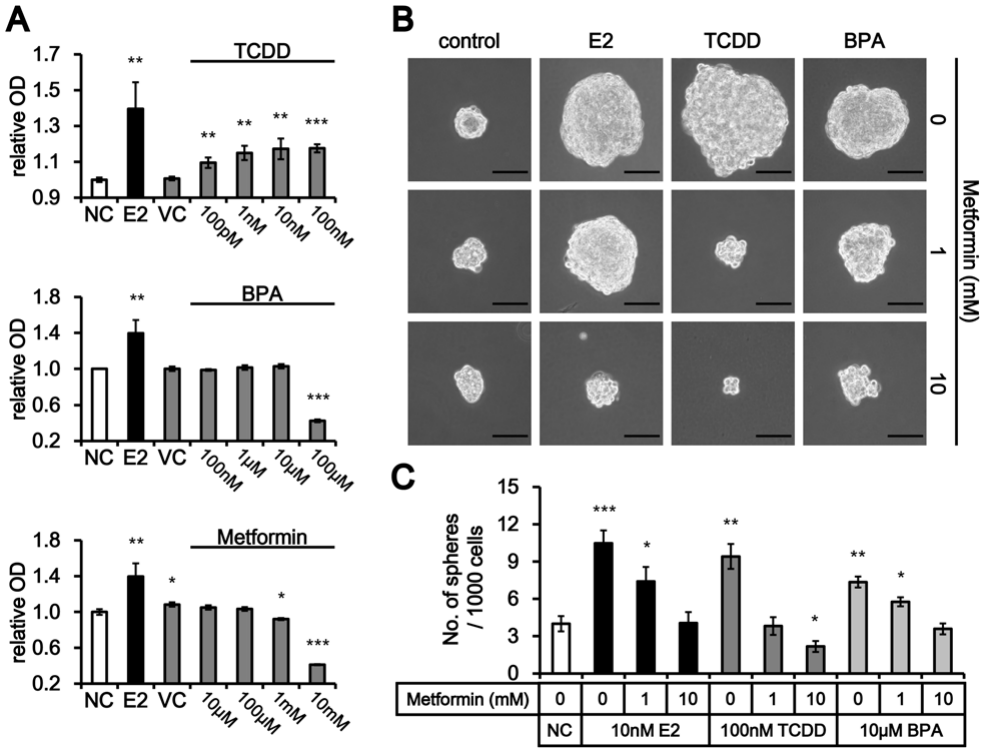
Regulation of MCF-7 mammosphere formation by breast cancer promoters and metformin. (A) TCDD increased proliferation of MCF-7 cells in a dose responsive manner (upper), however, BPA did not (middle). On the other hand, metformin treatment decreased MCF-7 proliferation (mean ± SD, n = 3). *, *P*<0.05; **, *P*<0.01; ***, *P*<0.001. (B, C) Metformin further decreased the size (B) and the number (C) of MCF-7 mammosphere formation enhanced by E2, TCDD or BPA (mean ± SD, n = 3). The magnification was X 200. Scale bar represents 50 µm in length. *, *P*<0.05; **, *P*<0.01; ***, *P*<0.001.

To know whether tumor promoting agents effect mammosphere formation, we used TCDD and BPA, both estrogenic disrupting chemicals, on MCF-7 mammosphere. On the other hand, metformin, an antidiabetic drug with anticancer effects against various diabetes-associated cancers, including breast cancer [3], [6], [31], was tested for regulation of mammosphere formation. MTT assay showed that TCDD increased MCF-7 cell proliferation in a dose dependent manner (Figure 5A upper), yet, the effect was lower than that of E2. BPA also increased MCF-7 cell proliferation up to 10 µM, however the increase was not statistically significant (Figure 5A middle). Metformin decreased MCF-7 cell growth at the 1 mM and 10 mM concentrations (Figure 5A lower). Lower doses of metformin than 1 mM did not show significant decrease in cell proliferation. To confirm the potential cytotoxicity, MTT assay was conducted only after a 24 h treatment. MCF-7 cells exhibited cytotoxicity at higher concentrations of BPA (>100 µM) but did not show cytotoxicity at 10 mM metformin (Figure S1).While the in vitro concentrations were higher than what is normally found in vivo, due to the complexity of in vitro-in vivo extrapolations [32], and the fact that the in vitro mammospheres were not vascularized, this difference might not be unexpected. Based on these results, we chose the 100 nM of TCDD and 10 µM of BPA, in which MCF-7 showed maximal enhancement of cell proliferation. In addition, 1 mM and 10 mM metformin were chosen for their inhibitory effects on MCF-7 cell growth. Efficiency of MCF-7 mammosphere formation was assessed after treatment of E2, TCDD or BPA with or without metformin treatment. As a result, the treatment of E2, TCDD and BPA without metformin increased the size of MCF-7 mammosphere (Figure 5B). Addition of the metformin exhibited reduction in sphere size. The numbers of mammospheres were significantly increased by treatment of the E2 and TCDD and metformin decreased the number of MCF-7 mammosphere in a dose dependent fashion (Figure 5C).

### The Control of ERE at the promoter region of OCT4

**Figure 6:**
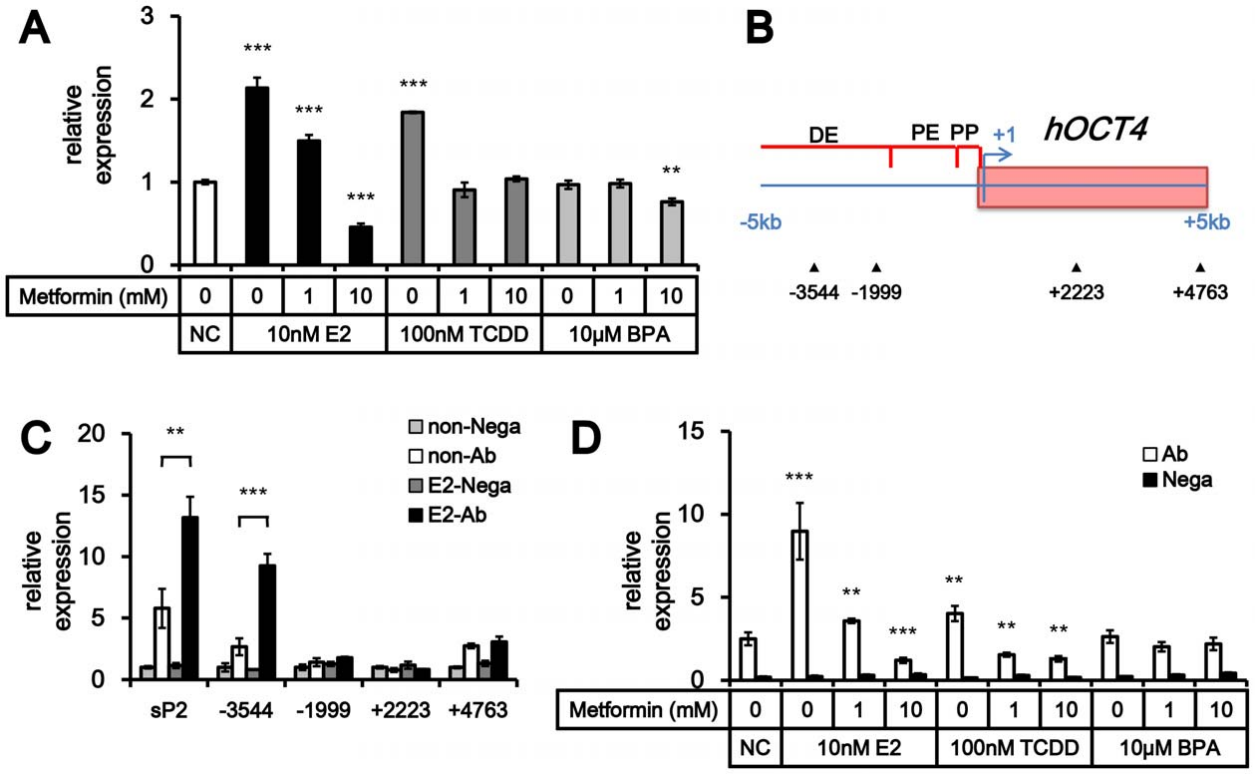
Regulation of OCT4 expression by metformin in MCF-7 cells. (A) E2 and TCDD increases OCT4 expression levels in MCF-7 cell line, however, BPA did not (mean ± SD, n = 3). **, *P*<0.01; ***, *P*<0.001. (B) Schematics of primer design for chromatin immunoprecipitation to detect putative ERE sequences in OCT4 promoter regions. Arrow heads indicate locations of putative ERE sequences. DE, distal enhancer; PE, proximal enhancer; PP, proximal promoter. (C) Chromatin immunoprecipitation to assess ER alpha binding at putative ERE sequences in OCT4 promoter region suggested that a putative ERE sequence at -3544kb from OCT4 transcription starting site was bound to ER alpha (mean ± SD, n = 3). **, *P*<0.01; ***, *P*<0.001. (D) The ERE sequence at -3544kb was enriched with ER alpha by treatment of E2 and TCDD compared to control and BPA treatment group. The enrichment was attenuated by co-treatment of metformin (mean ± SD, n = 3). *, *P*<0.05; **, *P*<0.01; ***, *P*<0.001.

We checked the *OCT4* expression level after treatment of E2, TCDD or BPA with or without metformin (Figure 6A). Interestingly, E2- and TCDD- treated cells, without metformin, showed increased expression level of OCT4, however, BPA did not. In addition, metformin blocked the enhancement of *OCT4* expression caused by treatment of E2 or TCDD in MCF-7 cells. On the other hand, BPA treatment in MCF-7 cells did not increase *OCT4* expression. To identify the role of estrogen signaling on OCT4 expression regulation, we searched estrogen binding elements (EREs) in the promoter region of *OCT4* gene. EREs are ER binding site highly conserved in several species [33]. Common ERE sequences (5′ – GGTCAnnnTGACC – 3′) are well known, and slight variations are acceptable [33]. We looked for common ERE sequences at the OCT4 promoter region, which ranges from 5 kb upstream to 5 kb downstream of transcription starting site, however, there was no identical sequences. We then screened sequences which have minor variation on 1 to 3 nucleotides. There were 4 ERE target sequence with minor sequence variations at *OCT4* promoter region (Figure 6B and Table S2). To identify whether the putative ERE binding sites are bound by ER, we performed ChIP assay (Figure 6C). pS2, which has well known ERE target sequence, was used as positive control. ChIP assay showed that binding of ER to a putative ERE binding site at -3544 kb of OCT4 transcription start site was enriched after treatment of E2 with similar level to that of pS2. Another putative binding site at +4763 kb showed a slight increase of ER binding, however, the enhancement of binding level was negligible. To confirm whether breast cancer promoting and inhibiting chemicals, we tried ChIP assay with treatment of TCDD, BPA and metformin in MCF-7 cells (Figure 6D). The binding of ER at OCT4 promoter showed similar patterns of OCT4 expression regulation. E2 and TCDD showed increased biding of ER on OCT4 promoter, however, BPA did not. The treatment of metformin decreased binding of ER on OCT4 promoter in E2 or TCDD treated cells.

## Discussion

Our studies were designed to determine if there might be a biological, non-cytotoxic mechanism to explain several epidemiological and experimental in vitro and in vivo studies, suggesting an anti-cancer effect of metformin. By using human breast cancer cell lines, with and without the estrogen receptor, grown in 3-dimension to try to mimic some in vivo conditions, we show that several known growth promoters or endocrine disruptors, 17-beta estradiol, phenol red, TCDD, and bisphenol A, did stimulate human breast cancer stem cells, as evidenced by both the number and sizes of MCF-7 mammospheres with the estrogen receptor and that metformin could suppress this stimulated MCF-7 cancer stem cell growth at non-cytotoxic concentrations.

The major findings of our studies demonstrated that (a) the 3-dimensional mammospheres could be used to detect both agents that stimulated or inhibited the numbers and growth of these estrogen receptor- positive human breast carcinoma cells; (b) that 17-beta estradiol, and the endocrine disruptors, phenol red, TCDD, bisphenol A, stimulated both the numbers and growth of the MCF-7 cancer stem cells; (c) that metformin could inhibit the mitogenic stimulus of estrogen and the endrocrine disruptors at non-cytotoxic concentrations; (d) that at low concentrations, estrogen stimulated the growth of the mammospheres, probably by an estrogen-dependent mitogenic signaling mechanism, whereas at higher concentrations, growth inhibition occurred, probably by some estrogen receptor -independent oxidative stress-induced mechanism, that blocked the estrogen-dependent signaling; (e) estrogen signaling increased OCT4 expression, while metformin interrupted estrogen-induced OCT4 expression; and (f) the mechanism by which bisphenol A enhanced MCF-7 cancer stem cell self-renewal, as evidenced by mammosphere numbers and growth were different than the mechanisms by which estrogen and TCDD worked.

One of the markers used to monitor the effect of both the estrogen and estrogenic-like compounds, as well as the effect of metformin, was the OCT4 gene. The *OCT4* gene is a member of POU family and functions as a transcription factor [34]. This gene is expressed in embryonic stem (ES) cells, germ cells [35], and adult human stem cells [36], while it helps to maintain an undifferentiated state and to prevent differentiation [37], [38]. In this study, we showed that *OCT4* expression can be induced by 10 nM 17-beta-estradiol in MCF-7 mammospheres. Usually estrogens act through two kinds of pathways, namely, an estrogen receptor-dependent pathway and an estrogen receptor-independent pathway in the cells [39].

Estrogens bind to the estrogen receptor of the nucleus to form ER-estrogen complexes in the ER-dependent pathway. These complexes might affect, directly, *OCT4* expression by binding to the *OCT4* gene promoter region, thereby, activating gene transcription. ER-estrogen complexes might also affect, indirectly, *OCT4* expression in relation to histone stability of *OCT4* gene promoter. When ER-estrogen complexes bind to the estrogen responsive element (ERE) of target genes, p160 and p300 are recruited to the ER-estrogen complexes and then the PBP/TRAP220/DRIP205 subunit interacts with complexes [40]. As these actions facilitate histone acetylation, the *OCT4* promoter region could be exposed to other transcription factors, thereby, inducing *OCT4* promoter activation. On the other hand, in the ER-independent pathway, estrogens might be metabolized to metabolites in cytoplasm. As a result, ROS are created. These ROS are the cause of oxidative stress. ROS induction of various intra-cellular signal transductors, for example, NF-κB, might be activated through this pathway [41]. Activated NF-κB could lead to histone deacetylase (HDAC) activation, inhibiting *OCT4* gene transcription. Recently, Itoh et al. reported that estrogen could dissociate physical incorporation of ER and HDAC2 which, in turn, could increase accessibility of ER-estrogen complex to promoter region of target genes [42]. Moreover, they reported that treatment of E2 increased transcriptional activity of Sp1, Sp3 transcription factors against GC- rich Sp1, Sp3 site in IL-1α promoter region. Given that Sp sites are also present in *OCT4* promoter region [43], it is reasonable to speculate that estrogen might affect *OCT4* gene transcription directly, or indirectly.

In this study, 17-beta-estradiol (E2) might affect *OCT4* expression through both pathways. In low concentrations, up to 20nM E2, the ER-dependent pathway might be activated to increase the *OCT4* expression and a mitogenic response. On the other hand, ROS production might be increased through an ER-independent pathway rather than the ER-dependent mechanism in high concentration 100 nM. The oxidative stress-induced signaling could inhibit the mitogenic signals of the estrogen-dependent pathway. Indeed, repression of *OCT4* expression in mammosphere treated with high concentration of E2 was seen. This suggests that ROS production, induced by 17-beta-estradiol metabolism, suppressed *OCT4* expression.

This dramatic increased expression of the *OCT4* after exposure of these cancer stem cells in mammospheres to estrogen suggests that estrogen stimulated the symmetrical cell proliferation of MCF-7 breast cancer stem cells. If this interpretation is correct, it becomes obvious that a potential human *in vitro* assay, using 3-dimenisional MCF-7 mammosphere culture, could be developed to screen for breast tumor promoters that might mimic what estrogen does to increase the expression of the *OCT4* gene and lead to human breast cancer.

The normal human breast stem cell expresses *OCT4*, does not express connexin43, and expresses the estrogen receptor, as well as other markers [44], [45], similar to the MCF-7 cells. Therefore, in order to “target” the human breast “cancer stem cells”, one must design new chemopreventive and therapeutic strategies that will affect the expression of the (a) *OCT4* (a gene needed to maintain the “stemness” of both the normal breast and breast “cancer” stem cells) and (b) *connexin 43* gene (a gene required for allowing differentiation to occur in the normal human breast stem cells). Rather than trying to “kill” tumor cells or even the “cancer stem cells”, altering the expression of these two genes could induce these “cancer stem cells” to terminally differentiate. However, up until now, there has been no systematic approach to screen for agents that might directly affect the *OCT4* and *connexin 43* genes, with the possible exception of the study with SAH [46]. Therefore, suspected human breast “carcinogens” could be detected by an increase of the expression of *OCT4* and estrogen receptor in the mammospheres, while continuing to suppress the expression of *connexin 43*.

The results of these studies, based on the use of *OCT4* as a normal or cancer stem cell marker and the three-dimensional mammospheres, implies many additional basic mechanistic experiments should be done to understand the biology of breast cancer stem cells and to screen for human breast tumor promoters and preventive/therapeutic agents for breast cancer. However, these studies do provide some mechanistic support for the epidemiological observations that metformin could be a useful anti-breast cancer chemopreventive treatment.

## Materials and Methods

### Cell lines

Human MCF-7 (ERα+, ERβ+) and MDA-MB-231 (ERα-, ERβ+) breast carcinoma cells were obtained from American Type Culture Collection (American Type Culture Collection, Manassas, VA). The Human SK-BR-3 (ERα-, ERβ-) breast cancer cells were obtained from Korea Cell Line Bank (Korea Cell Line Bank, Seoul, Korea). Transformation of normal human breast epithelial cells, Type I HBEC, was achieved by introduction of SV40 DNA as previously described [44]. The SV40-transformed immortal Type I HBEC derived cell line (M13SV1) is non-tumorigenic, but X-ray-radiated M13SV1 cell line (M13SV1 R2) is weakly tumorigenic and neu oncogene-transfected M13SV1 cell line (M13SV1 R2N1) is highly tumorigenic [44].

### Chemicals

To test estrogen and anti-estrogen effects on MCF-7 mammosphere formation, 17-beta-estradiol (Sigma Chemical Co., Saint Louis, MO) and potent ER inhibitor ICI182,780 (Sigma Chemical Co) were used. An antioxidant N-acetyl-L-cysteine (NAC, Sigma Chemical Co) was used to test whether oxidative stress caused by high concentration of estrogen affects mammosphere formation. 2,3,7,8,-tetrachlorodibenzo-p-dioxin (TCDD), bisphenol A (BPA) and metformin were also purchased from Sigma Chemical Co.

### Preparation of Mammospheres

Single cells were plated in ultralow attachment plates (Corning Costar Corp., Cambridge, MA) at a density of 10,000 viable cells/ml in primary culture and 1000 cells/ml in subsequent passages. Cells were grown in a serum-free mammary epithelial basal medium (MEBM, Cambrex Bio Science Inc, Walkersville, MD), supplemented with 2% B27 (Invitrogen, Carlsbad, CA), 20 ng/ml EGF (BD Biosciences, Franklin Lakes, NJ), antibiotic-antimycotic (100 unit/ml penicillin G sodium, 100 µg/ml streptomycin sulfate and 0.25 µg/ml amphotericin B) (Invitrogen), 20 µg/ml Gentamycin, 1 ng/ml Hydrocortisone, 5 µg/ml Insulin and 100 µM 2-mercaptoethanol (Invitrogen) in a humidified incubator.

### FACS analysis

By using a FACSAria (Becton Dickinson, San Jose, CA), the expression of a panel of breast cancer stem cell markers was distinctly evaluated on cells obtained from mammospheres. The antibodies used were phycoerythrin (PE)-conjugated anti-CD24 and fluorescein isothiocyanate (FITC)-conjugated anti-CD44 (BD Pharmingen, San Diego, CA). Staining was done according to the instructions of the manufacturer.

### Immunocytochemistry

Mammospheres attached to 4-chamber slides were fixed immediately in 4% paraformaldehyde and permeabilized 0.4% Triton X-100 for 20 minutes. Mammospheres were blocked with 10% normal goat serum (Zymed Laboratories Inc, San Francisco, CA) at 4°C overnight and then incubated with rabbit anti-OCT4 polyclonal antibody (Chemicon, Temecula, CA), followed by incubation with an Alexafluor 594-labeled goat anti-rabbit IgG (Invitrogen). Nuclear staining was performed by 4′,6-diamidino-2-phenylindole (DAPI, Sigma Chemical Co.). Images were captured on a Nikon C1si spectral imaging confocal system (Nikon, Tokyo, Japan).

### Semi-quantitative and real-time quantitative reverse transcription-polymerase chain reaction (RT-PCR) analysis

Total cellular RNA was extracted from cells by using TRIzol reagent^TM^ (Invitrogen). cDNA synthesis was accomplished by adding the purified RNA and oligo-dT primers to Accupower RT premix (Bioneer, Daejeon, Korea). PCR was conducted with Accupower PCR premix (Bioneer). All procedures were done as described by manufactures. Primers for human *OCT4* amplification were made based on a literature [47]. Realtime-PCR was performed by mixing cDNA with Power SYBR Green PCR Master Mix (Applied Biosystems, Foster City, CA) with an ABI 7500 Realtime-PCR System (Applied Biosystems) according to the manufacturer's instructions. Gene expression levels were compared after normalization to endogenous GAPDH. The primer sequences used in this study are illustrated in Table S1.

### MTT cell proliferation assay

The proliferation potential of cells was measured using the MTT assay, which is based on the ability of live cells to convert tetrazolium salt into purple formazan. Briefly, cells (1×10^4^ cells per well) were seeded in 24-well microplates in 450 µl media. After 48 h, 50 µl MTT stock solution (5 mg/ml, Sigma Chemical Co) was added to each well, and the plates were further incubated for 4 h at 37°C. The supernatant was removed, and 200 µl DMSO was added to each well to solubilize the water insoluble purple formazan crystals. The absorbance at a wavelength of 540 nm was measured with an EL800 microplate reader (BIO-TEK Instruments, Winooski, VT).

### ROS detection

ROS detection was performed according to the manufacturer's protocol (Invitrogen). Briefly, MCF-7 mammospheres were gently washed with PBS before staining. After that, the mammospheres were incubated with 25 µM carboxy-H2DCFDA for 30 min at 37°C, protected from light. Spheres were washed three times with PBS. Carboxy-DCF was detected by confocal microscope at 495/529 nm.

### ChIP and luciferase reporter assays

ChIP assays were performed according to the manufacturer's protocol (Upstate Biotechnology, Waltham, MA). Chromatin was immunoprecipitated using rabbit anti-human ERα antibodies (sc-8002, Santacruz). PCR was performed at a final template dilution of 1∶50. The primer sequences used in this study are supplied in Table S2.

### Statistical analysis

The data were expressed as the mean plus or minus the standard error. Analyses were performed using computerized statistical software. Statistically significant (*P*<0.05) data were further analyzed by Dunnet's t-tests.

## Supporting Information

Figure S1 **Cytotoxicity of TCDD, BPA, or metformin.** (A–C) MTT assay for 24 h treatment of TCDD, BPA, or metformin in MCF-7 cells. Only 100 µM BPA showed cytotoxicity (mean ± SD, n = 3). ***, *P*<0.001.
(TIF)

Table S1 **Primer sequences used for RT-PCR.**
(DOC)

Table S2 **Primer sequences used for chromatin immunoprecipitation assay for putative estrogen binding sites.**
(DOC)
