# Supplementary figures and images for: Metformin Represses Self-Renewal of the Human Breast Carcinoma Stem Cells via Inhibition of Estrogen Receptor-Mediated OCT4 Expression
Source: PLoS One. 2011 Nov 23;6(11):e28068. doi: 10.1371/journal.pone.0028068 (PMC3223228; doi:10.1371/journal.pone.0028068)

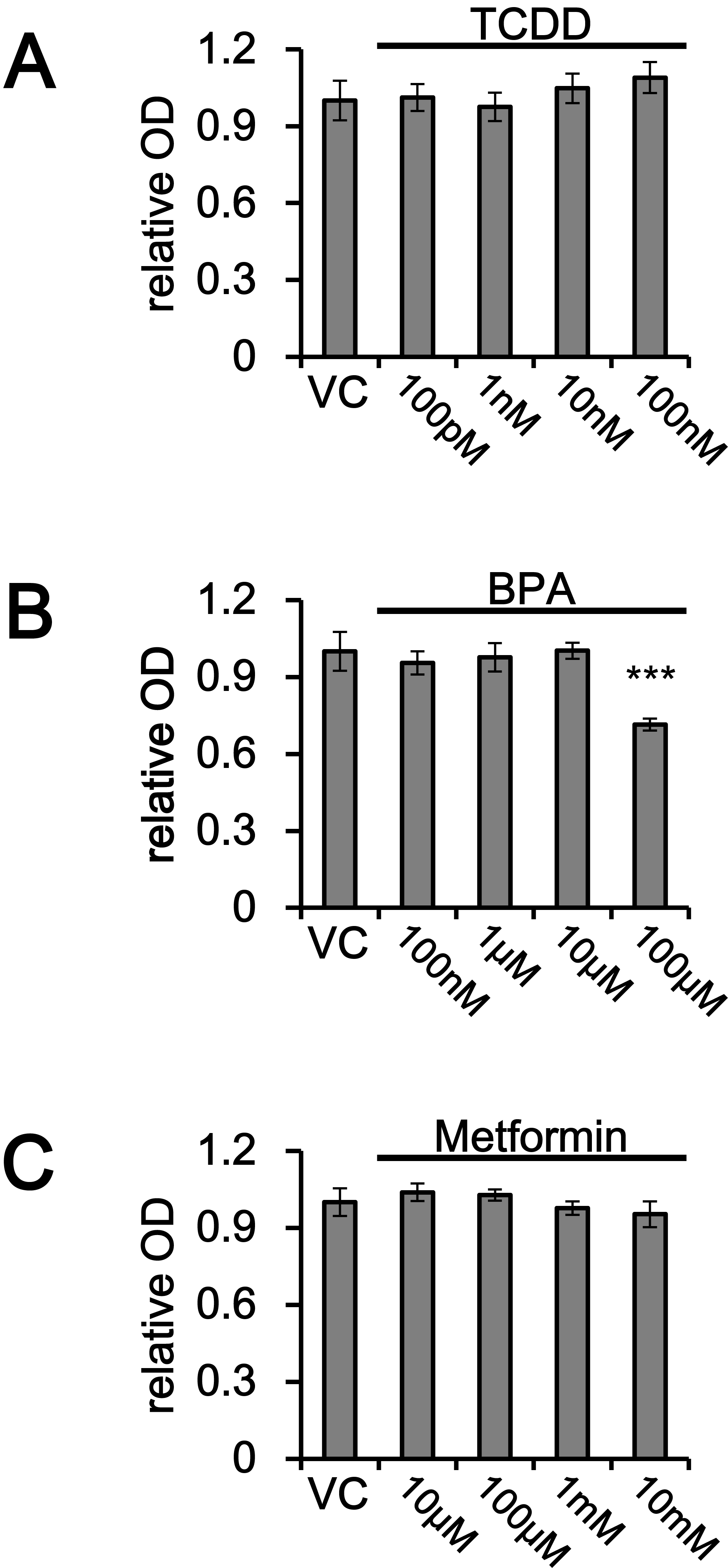

Supplement: Figure S1 — Cytotoxicity of TCDD, BPA, or metformin. (A–C) MTT assay for 24 h treatment of TCDD, BPA, or metformin in MCF-7 cells. Only 100 µM BPA showed cytotoxicity (mean ± SD, n = 3). ***, P<0.001. (TIF) [file pone.0028068.s001.tif]
