# Supplementary material for: Metformin Represses Self-Renewal of the Human Breast Carcinoma Stem Cells via Inhibition of Estrogen Receptor-Mediated OCT4 Expression
Source: PLoS One. 2011 Nov 23;6(11):e28068. doi: 10.1371/journal.pone.0028068 (PMC3223228; doi:10.1371/journal.pone.0028068)
Supplement: Table S1 — Primer sequences used for RT-PCR. (DOC) [file pone.0028068.s002.doc]

| **Primer** | **Sequences** |
| --- | --- |
| ***OCT4*** | <F> 5' – GACAACAATGAAAATCTTCAGGAGA – 3' |
|  | <R> 5' – CTGGCGCCGGTTACAGAACCA – 3' |
| ***GAPDH*** | <F> 5' – CATGAGAAGTATGACAACAGCCT – 3' |
|  | <R> 5' – AGTCCTTCCACGATACCAAAGT – 3' |

**Table S1.** Primer sequences used for RT-PCR.
