# Supplementary material for: Metformin Represses Self-Renewal of the Human Breast Carcinoma Stem Cells via Inhibition of Estrogen Receptor-Mediated OCT4 Expression
Source: PLoS One. 2011 Nov 23;6(11):e28068. doi: 10.1371/journal.pone.0028068 (PMC3223228; doi:10.1371/journal.pone.0028068)
Supplement: Table S2 — Primer sequences used for chromatin immunoprecipitation assay for putative estrogen binding sites. (DOC) [file pone.0028068.s003.doc]

**Table S2.** Primer sequences used for chromatin immunoprecipitation assay for putative estrogen binding sites.

| **Primer** | **Sequences** |
| --- | --- |
| -418 pS2 promoter | <F> 5' – CCTGCAAGGTCACGGTGGCC – 3' |
|  | <R> 5' – GGCCCTCCCGCCAGGGTAAA – 3' |
| -3544 OCT4 promoter | <F> 5' – TCCTCCCAGCTCACCCACTCC – 3' |
|  | <R> 5' – TCTCCCCCATGAGCCCTGCA – 3' |
| -1999 OCT4 promoter | <F> 5' – GACAGCTGGCCACGGGACAC – 3' |
|  | <R> 5' – AGGCCAGGTCTGGACTGGGC – 3' |
| -2223 OCT4 promoter | <F> 5' – CTTCCTCCCTACTGTCTGTGGCC – 3' |
|  | <R> 5' – ACAGGCATGCGTCACCACGC – 3' |
| -4763 OCT4 promoter | <F> 5' – CCCTCTGCAGATTCTGACCGCA – 3' |
|  | <R> 5' – CCACCTCTTCCCAGAGGGAGCTC – 3' |
